# Working Collaboratively: Outcomes of Geriatrician Input in Older Patients Undergoing Emergency Laparotomy in a District General Hospital

**DOI:** 10.7759/cureus.7069

**Published:** 2020-02-21

**Authors:** Kashuf A Khan, Thejasvi Subramanian, Megan Richters, Ayesha Mubarik, Abdalla Saad Abdalla Al-Zawi, Christopher C Thorn, Susan Chalstrey, Savithri Gunasekera

**Affiliations:** 1 General Surgery, Royal Shrewsbury and Telford National Health Service (NHS) Trust, Shrewsbury, GBR; 2 General Surgery, Good Hope Hospital, University Hospitals Birmingham, Birmingham, GBR; 3 Internal Medicine, Great Western Hospital National Health Service (NHS) Foundation Trust, Swindon, GBR; 4 Family Medicine, Whiston Hospital, St Helens and Knowsley Teaching Hospitals National Health Service (NHS) Trust, Rochdale, GBR; 5 General Surgery, Mid and North Essex University Hospital Group, Basildon, GBR; 6 General Surgery, Great Western Hospital National Health Service (NHS) Foundation Trust, Swindon, GBR; 7 Otolaryngology, Great Western Hospital National Health Service (NHS) Foundation Trust, Swindon, GBR; 8 Geriatrics, Frimley Health National Health Service (NHS) Foundation Trust, Frimley, GBR

**Keywords:** geriatric surgical liaison, emergency laparotomy, length of stay, perioperative care, ageing & frailty, cepod, cognitive impairment, elderly patients

## Abstract

With the increasing median age of survival in the UK, there is an increased burden on the provision of medical and surgical care to the population. The 2010 National Confidential Enquiry into Patient Outcome and Death report, “An Age Old Problem,” emphasizes the early involvement of surgical and geriatric consultant input to improve perioperative care in older patients.

This study describes the development of a Geriatric Surgical Liaison Service aimed at providing consultant-led geriatrician support to improve the outcomes of older patients undergoing Emergency Laparotomy (EL). The primary outcome is the reduction in length of stay (LOS) compared to baseline data prior to geriatrician involvement.

The service was designed to include one clinical session involving a consultant geriatrician and two and a half days with a junior doctor in a week. Data was collected prospectively from February 2018 till July 2018 for surgical patients aged ≥ 70 years, who underwent EL, had an inpatient stay of more than seven days, and who were diagnosed with delirium or incurred inpatient falls (intervention group). Baseline data, prior to geriatrician involvement, were collected retrospectively for EL patients aged ≥ 70 years from December 2015 until May 2016. Length of stay and 30-day mortality were also compared between the two cohorts undergoing EL.

A total of 69 patients were included in the intervention group; 45 patients underwent EL and their mean LOS was 17.5 days, which was reduced from 22.5 days prior to geriatrician involvement (n=57). There was no difference in median length of stay and 30-day mortality between the retrospective baseline group and the intervention groups. In the intervention group, 8.5% of patients had a new medical diagnosis and 26.8% of patients were offered follow-ups.

Although statistically not significant (p=0.40), a shorter stay in hospital by five days can potentially have a positive impact on patient outcomes by reducing psychosocial, cognitive, and functional deconditioning. This would also improve patient flow, release capacity, and waiting times and would be of benefit to the financially strained National Health Service (NHS). Overall, our study suggests that a collaborative, consultant-led geriatric service can improve the management of older surgical patients by potentially reducing length of stay, identifying high-risk patients, and facilitating early and appropriate specialty input alongside adequate and required outpatient follow-up.

## Introduction

The population in the UK is aging; with approximately 8% of the population aged over 75 years. Surgery in this patient group accounts for 23% of all surgical procedures performed in the UK [1]. The increasing number of older patients undergoing surgery poses a unique and difficult challenge to the surgeon and the health service in general due to increased morbidity and mortality, reduced physiological reserve, and increased risk of cognitive impairment associated with aging and frailty in this ever-increasing patient cohort [2].

Although only 11% of hospital admissions account for emergency procedures, they are responsible for 50% of all in-hospital morbidity and mortality [3-4]. Older patients requiring emergency surgery are most sensitive to disruptions in their physiological and cognitive baselines, such as acute fluid and electrolyte disturbances associated with sepsis or third-space fluid losses. Dealing with their surgical condition on the background of medical complexities warrants skilled resuscitation and vigilant monitoring throughout the perioperative period with prompt access to specialist recommendations as required [5].

In the UK, surgical trainees often manage older patients undergoing elective and emergency surgery, however, formal training is often inadequate and unstructured. This is unlike surgery in the pediatric population, which is part of the higher surgical training curriculum [6]. A trainee survey performed in 2013 revealed 90% of surgical trainees supported the inclusion of geriatric medicine teaching in the surgical curriculum to improve training, education, and support around the perioperative management of complex older patients [7].

The 2010 National Confidential Enquiry into Patient Outcome and Death (CEPOD) report, “An Age Old Problem,” emphasizes the early involvement of surgical and geriatric consultant input to improve perioperative care in the elderly. The report also recommends improving the education and training of geriatricians, anesthetists, and surgeons to aid early recognition of high-risk patients and providing early, effective management [8]. Previous liaison models of care (i.e., orthogeriatrics) have shown collaboration between surgeons and geriatricians to be beneficial in improving patient care; however, current provision in general surgery is lacking [7].

In view of this critical need, our Trust developed a Geriatric Surgical Liaison Service, with support from the postgraduate medical education department. This service was aimed at providing consultant-led geriatric support to improve the outcomes of older patients undergoing emergency general surgery.

The aim of our study was to assess the logistics of service provision, its effect on comparable outcomes before and after intervention, and understand the challenges encountered when introducing a change into a traditionally run ward and clinical care model.

## Materials and methods

A Liaison Geriatric Service was constituted of one clinical session involving a consultant geriatrician and two and a half days per week with a clinical innovation fellow (CIF) who had a background in general surgery and geriatrics. The CIF (a clinician at the level of junior core surgical trainee) was responsible for older patient recruitment into the service and acted as a liaison between the surgical team and the consultant geriatrician. Twice weekly, consultant-led ward rounds were performed with the parent team of surgical junior doctors, nursing, and allied health professionals implementing the management plans suggested.

Inclusion criteria for older patients eligible for the intervention included any patient undergoing an Emergency Laparotomy (EL) aged 70 years or older, and any patient aged 70 years or older with inpatient stay exceeding seven days. Surgical patients with diagnosed delirium by the parent team or who sustained an inpatient fall were also included in the study group. Patient recruitment was carried out from the emergency theater database and a referrals book was kept on the surgical ward with the inclusion criteria made clear to incoming junior surgical doctors, registrars, and consultants during regular MDT meetings.

All data was collected prospectively over a six-month period between February 2018 and July 2018. Patient demographics, comorbidities, indications, and the procedure at emergency laparotomy were recorded from the theater database, along with any postoperative complications. Date of geriatric review, subsequent investigations, further interventions, discharge disposition, and new diagnoses were recorded. Finally, morbidity, 30-day mortality, and total length of stay (LOS) were also recorded.

For the comparison, baseline data was collected by doing a retrospective study of all patients who had undergone an emergency laparotomy aged 70 years or older from December 2015 till May 2016. This was prior to the introduction of a geriatrician. LOS, 30-day mortality, and discharge destination were compared between the two data sets.

The primary aim was a reduction in LOS following a geriatric-led service intervention. Secondary parameters included the optimization of pain management, rehabilitation, end of life (EOL)/treatment escalation plan (TEP) discussion and implementation, medical optimization, new diagnosis, and follow-up in the prospective cohort. Statistical comparison was made using Microsoft Excel (Microsoft Corporation, Redmond, Washington) and StatPlus (AnalystSoft, Alexandria, VA) with parametric data compared with the student’s t-test and non-parametric data compared using the Mann-Whitney U test or the chi-square test. p-value <0.05 was considered statistically significant.

## Results

In the baseline cohort between December 2015 and May 2016, 57 patients underwent emergency laparotomy with an average age of 81.2 years. Median and mean LOS was 18 days and 22 days, respectively, with the majority of patients discharged back home as opposed to residential/nursing homes or rehabilitation wards (74% vs 19%). Four patients passed away within 30 days of emergency laparotomy (7%) (Table 1).

**Table 1:** Comparison between retrospective and prospective cohorts (* length of stay, **nursing home/rehabilitation/residential home) † student’s t-test; Ω Mann-Whitney U-test; ∆ chi-squared test

	Retrospective	Prospective (all patients)	Prospective (emergency laparotomy patients)	Comparing Retrospective + Prospective Patients (p-value)	Comparing Retrospective + Emergency Laparotomy Patients (p-value)
	Dec 15 - May 16	Feb 18 - Jul 18	Feb 18 - Jul 18		
No. of patients	57	69	45		
Mean age (years)	81.2 (71-94)	80.1 (69-96)	80.2 (70-96)	0.36^†^	0.43^†^
Median LOS* (days)	18 (2 - 92)	16 (5-41)	16 (8-41)	0.23^Ω^	0.40^ Ω^
Mean LOS* (days)	22.4	17.4	17.8	0.23^Ω^	0.40^ Ω^
No. of patients sent home (%)	42 (74%)	47 (66%)	33 (70%)	0.49^∆^	0.97^∆^
No. of patients sent NH/REHAB/RH**	11 (19%)	13 (18%)	8 (17%)		
30-day mortality	4 (7%)	7 (10%)	4 (8.5%)	0.54	0.82^∆^

Sixty-nine patients were included in the intervention period cohort between February 2018 and July 2018. Forty-five patients underwent emergency laparotomy (EL). Twenty-two patients had other surgical management not involving an emergency laparotomy (non-EL) and two patients underwent elective procedures - these patients also met inclusion criteria because of LOS of more than seven days. All patients eligible for the service were reviewed by a consultant geriatrician. EL patients were reviewed at a median of three days postoperatively (0-12 days). Non-EL patients were generally reviewed later at a median of 1.5 days (4-28 days).

There was no difference in the mean patient age, the median length of stay, and 30-day mortality between the retrospective baseline group and the intervention groups (Table 1). There was also no difference in the discharge destination between the baseline and intervention cohort. The mean length of stay was reduced in the intervention group by five days though this was not statistically significant (22.5 vs 17.5 days, p= 0.40).

**Figure 1:**
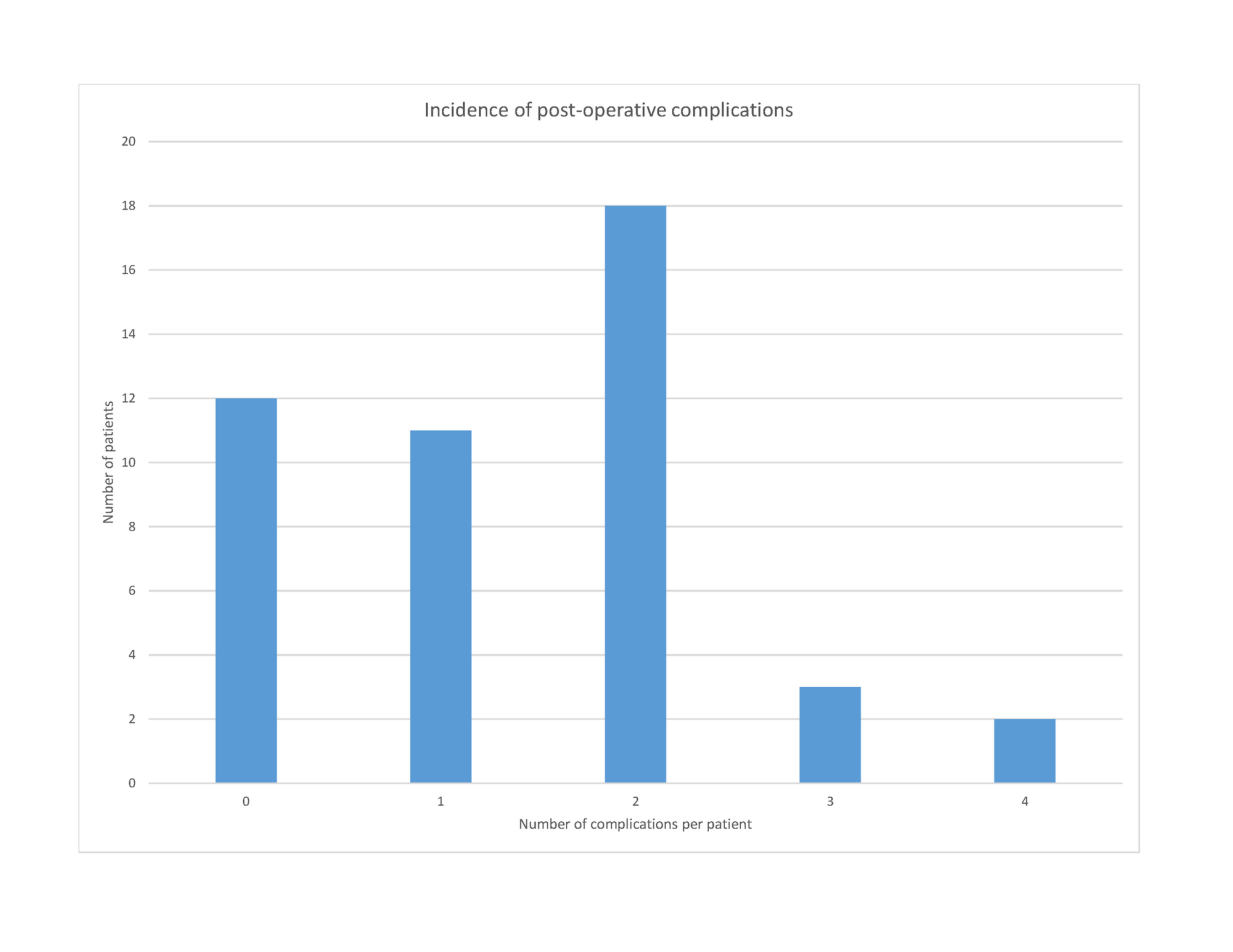
Incidence of postoperative complications in emergency laparotomy patients

The most common procedure performed during emergency laparotomy was Hartmanns, followed by adhesiolysis for patients with small bowel obstruction and small bowel resections for perforation (Table 2). Twelve patients (26.7%) who underwent an emergency laparotomy had uncomplicated postoperative recoveries. Thirty-three EL patients (73.3%) developed complications postoperatively (Table 3). Most patients developed two complications postoperatively, with an average of 1.42 complications per patient (Figure 1). Two patients required returns to the theater: exploration of laparotomy wound and a re-look laparotomy.

**Table 2:** Breakdown of emergency laparotomy procedures and surgical findings

Emergency Laparotomy Procedure	Numbers	Surgical Pathology
Hartmanns	11	Perforation (6)
		Malignancy - Sigmoid (4)
		Sigmoid volvulus
Adhesiolysis	9	Adhesional small bowel obstruction
Small bowel resection	6	Perforation (5)
		Small bowel obstruction
Loop colostomy	4	Large bowel obstruction (2)
		Malignancy - rectal (2)
Appendicectomy	2	Appendiceal abscess
		Perforation
Small bowel resection + hernia repair	2	Hernia - parastomal
Loop Ileostomy	2	Femoral hernia
Loop Ileostomy hernia repair	2 2	Bladder cancer
		Hernia - incisional
Hernia repair Small bowel resection + hernia repair + ileostomy	2 1	Hernia - umbilical
		Hernia - inguinal
Laparotomy	1	Hemodynamic instability post-lap R hemi
Enterolithotomy	1	Gallstone ileus
Right hemicolectomy	1	Malignancy - cecum
Right hemicolectomy + ileostomy	1	Malignancy - ascending colon
Splenectomy	1	Splenic bleed
Subtotal colectomy + end ileostomy	1	Colitis

**Table 3:** Breakdown of postoperative complications

Emergency Laparotomy Complication Systems	Numbers	Specific Complications
Neurological	16	Delirium (15)
		Stroke (1)
Cardiovascular	13	Atrial fibrillation (10)
		Fluid overload
		Myocardial infarction
		Heart block
Renal	10	Acute kidney injury (9)
		Hydronephrosis related to pelvic mass
Respiratory	8	Hospital-acquired pneumonia (5)
		Pneumothorax (2)
		Pleural effusion
Wound-related	7	Infection (5)
		Dehiscence (2)
Gastrointestinal	6	Ileus (5)
		C. difficile
Miscellaneous	3	End of life
		Gram-negative sepsis
		Neutropenia

Seven non-EL patients underwent surgery. Two patients underwent loop colostomies for a low rectal malignancy and rectal prolapse. Two patients had percutaneous endoscopic gastrostomy (PEG) insertions for gastric volvulus and post-PEG insertion bleed. Three patients had elective colorectal resection procedures, including an elective laparoscopic right hemicolectomy for a cecal malignancy, an elective panproctocolectomy (for colitis), an anterior resection, and ileostomy formation for rectal malignancy.

With regards to patient co-morbidities, only four patients of the 69 had no past medical history documented. Sixteen patients (23.2%) had fewer than three co-morbidities. Thirty patients (43.5%) had between three and six co-morbidities and 19 patients (27.5%) had more than six co-morbidities. The most common group of comorbidities were cardiovascular, endocrine, and gastrointestinal, respectively. Twenty-nine patients (42%) had hypertension, 16 patients (23.1%) had type two diabetes mellitus, and 15 (21.7%) had a history of ischaemic heart disease. Twelve patients (17.4%) suffered from chronic kidney disease and 11 patients were diagnosed with diverticulosis in the past. Nineteen patients had previous abdominal surgery though not all of the procedures were documented for all patients.

Consultant geriatrician review in the intervention group resulted in 39 patients (56.5%) having their medications reviewed (Table 4). Seventeen medications were adjusted under the guidance of the consultant geriatrician including antihypertensives (seven) and anticoagulant medications (four) were adjusted following the review. Steroid (two), metronidazole (one), benzodiazepine (one), antipsychotic (one), and potassium supplement (one) prescriptions were also adjusted.

**Table 4:** Clinical input from geriatrician following review *physiotherapy (PT)/occupational therapy (OT), **treatment escalation plan (TEP)/advance care planning (ACP)

Clinical input from Geriatrician	All Patients	Percentage	Emergency Laparotomy Patients	Non-Emergency Laparotomy Patients
Analgesia reviewed and adjusted	4	5.8%	2	2
PT/OT* input recommended	12	17.4%	9	2
TEP Completed prior to review	16	23.2%	8	7
TEP/ACP** Initiated	6	8.7%	4	1
Medication review	39	56.5%	19	20
Glycemic control optimized	4	5.6%	1	3

Twenty-seven patients (39.1%) had further investigations requested following the geriatric review. Bedside investigations included orthostatic blood pressure, arterial blood gas, urine osmolality/red blood cell (RBC) casts, and urine protein creatinine ratio. Specific blood tests were requested in eight patients (11.3% of patients) mainly for monitoring electrolyte imbalances, performing confusion screens, or checking B12/folic acid and ferritin levels. Further imaging was advised in 10 patients, including staging computed tomography (CT), chest X-ray (three), CT brain (three), and ultrasound urinary tract (Table 5).

**Table 5:** Breakdown of further investigations following by geriatrician input ECG: electrocardiography

Further intervention Advised by Geriatrician		All Patients	Percentage of All Patients	Emergency Laparotomy Patients	Non-Emergency Laparotomy Patients
Further investigations requested	Bedside investigations	6	8.5%	2	4
	Extra blood tests	8	11.3%	3	5
	48 hours ECG tape	9	14.1%	8	1
	Echo	6	8.5%	5	1
	Further imaging	10	14.1%	5	5
	Other	1	1.4%	0	1
New diagnosis		5	8.5%	2	3
No. of referrals to other specialties		24	33.8%	10	13
Outpatient follow-up with other specialties		19	26.8%	13	6

Following the intervention, 21 patients were referred to other specialties, as inpatients with three patients being referred to more than one specialty. Five patients were referred to cardiology, with one patient requiring angiography and percutaneous intervention for a presumed acute coronary syndrome. Four patients were referred to the inpatient mental health liaison. Two patients were referred to palliative care, rheumatology, and hematology each. Other inpatient referrals included other medical and surgical specialties (stroke, renal, gastroenterology, orthopedics, ear, nose, and throat (ENT), and so on) but also allied health professionals such as speech and language therapists and chest physiotherapy.

Additionally, 15 patients had symptoms warranting further follow-up organized as an outpatient: seven patients in the virtual clinic, five in the memory clinic, and others in falls, orthopedic, hematology, respiratory, and endocrinology.

## Discussion

Overall, our study suggests that an organized and collaborative consultant-led geriatric service can improve the management of older surgical patients by potentially reducing LOS, identifying high-risk patients, and facilitating early and appropriate specialty input alongside adequate and required outpatient follow-up.

Reduction in length of stay

Mean and median LOS were reduced in the intervention group following input from a single consultant geriatrician. Although not statistically significant (p = 0.40), mean LOS was reduced by five days per patient, suggesting that improvement is possible. A recent study by Shipway et al. reported a mean 3.1 days (10.6 vs 7.5 days, p = 0.007) reduced LOS in all patients over 60 years of age undergoing gastrointestinal (GI) surgery following the establishment of a geriatric liaison service [9]. The study included robust preoperative assessment clinics, comprehensive geriatric assessments, twice-weekly geriatric ward rounds, and multidisciplinary team meetings and access to a geriatrician-led surgical rehabilitation ward. Notably, patients over 75 years of age undergoing emergency gastrointestinal surgery had a mean reduction in LOS of 2.75 days (which was not statistically significant (9.0 vs 6.3 days, p=0.091), similar to our findings. Nevertheless, the study highlights the potential of systematic service development in reducing prolonged hospital stay - an outcome that has been shown to significantly affect muscle mass, functional ability, and risk of postoperative complications [10-11].

Complications, multimorbidity, and interventions

Patients in our study had a wide array of comorbidities prior to hospitalization, with 42.3% of patients having up to six co-morbidities. As a result, they were at a higher risk of complications affecting different physiological systems, warranting reviews from individual specialties. This further validates that a review of such patients by a geriatrician focusing on the proactive inpatient management of complications and comorbidity. The consultant geriatrician is able to provide general, holistic care as well as advise prompt referrals to pertinent specialties rather than review systems in isolation [12]. This can be seen in the number of new diagnoses made as an inpatient (8.5% of all patients) and, arguably, more importantly, the number of patients who have been referred for outpatient follow-up appointments upon discharge (26.8%). Additionally, regular geriatrician input provides the patient and the parent team a fresh perspective of the clinical journey of the postoperative patient. Of note, we observed more opportunities were taken to conduct meaningful discussions about end-of-life decisions and ceilings of care, which were not previously addressed. It is unlikely that the breadth of medical pathology can be addressed by a general surgical team in a complex multi-morbid patient population during a vulnerable perioperative period warranting the role of a regular geriatric review of such a patient cohort.

Positive cultural change and buy-in from the surgical team

Introducing change in any established system is challenging. In order to maximize the benefits of the service, the support and ownership of the surgical team were necessary. The provision of a CIF with a background in surgery provided the bridge between the two teams - allowing it to work effectively. At the beginning of the service, few referrals were made by the surgical team but this significantly increased after including an introduction to the new service at the junior doctor surgical induction. Alongside increased awareness and encouragement by the surgical consultants, more referrals were made, resulting in more patients being considered for review and, subsequently, improved perioperative management.

Economic viability

One consultant geriatrician clinical session and 50% of CIF time were allocated each week for the provision of service. Post Graduate Medical Education funded the 50% CIF time - approximately £9,115 over six months. Other staffing (nursing, physiotherapy, occupational therapy, and so on) was drawn from existing resources and no additional investment was required. In 2017/2018, the national tariff for per day stay (for day exceeding trim point) for complex abdominal surgeries was £222 per patient [13]. This suggests an annual saving of approximately £111,000 per 100 patients. Although the cost of referrals to other specialties, investigations, and outpatient appointments were not calculated, we believe that the cost of the consultant geriatrician's time and subsequent liaison team was justified despite the financial constraints and may prove to provide further benefits for later resource allocation.

Limitations

Ours is a single-center pilot study showing early results from a small number of patients. This study was inadequately powered due to a limitation in the patient pool. We need more studies like this on a broader patient population to potentially show statistically significant outcomes. There are variations between the retrospective and prospective cohorts with retrospective data collection, including only patients aged ≥70 years who underwent an emergency laparotomy - a different cohort compared to patients under review in the prospective study. In addition, there is no data available on comorbidities for this group to establish any comparisons in terms of frailty or preoperative morbidity which may affect LOS, complications, and mortality rate.

We acknowledge the role of Comprehensive Geriatric Assessments (CGAs), preoperative clinics, and optimization; however, our resources did not allow us to establish such a service. We hope that the results of this six-month trial will encourage effective resource and time allocation in the future.

Frailty is a compelling indicator of mortality, LOS, and readmission within 30 days and it has been recognized as a distinct entity from increasing age alone [14-16]. Studies have shown that one-fifth of older adults and 16% of younger adults presented with surgical emergencies are deemed to have frailty [17-18]. It may be that a frailty assessment on admission proves more beneficial at identifying patients in need of additional care than age.

To ensure continuity of care, we recommend a perioperative care fellow to be appointed to liaise with current provision. Current recommendations also suggest that improved outcomes for older patients in the perioperative period are associated with the engagement of a geriatrician [7,19].

## Conclusions

Our study did not result in a statistically significant reduction in LOS or return to the previous living arrangement following a consultant geriatrician's input of older patients undergoing emergency laparotomy surgery. However, we have noted the valuable contribution of a consultant geriatrician in orchestrating rehabilitation, performing medication reviews, conducting end-of-life discussions, optimizing pain management, and diagnosing, investigating, and following up on new pathology in the perioperative older patient. This represents a proactive and comprehensive model of care, which, with further investment and research, may result in a change in working culture and, ultimately, improvement in patient care.
